# The Relationship between Inter-Arm Blood Pressure Difference and Coronary Artery Disease Severity Calculated by the SYNTAX Score

**DOI:** 10.1155/2018/9370417

**Published:** 2018-09-13

**Authors:** Gündüz Durmuş, Erdal Belen, Akif Bayyigit, Muhsin Kalyoncuoğlu, Mehmet Mustafa Can

**Affiliations:** ^1^Department of Cardiology, Haseki Education and Research Hospital, Istanbul, Turkey; ^2^Department of Internal Medicine, Okmeydanı Education and Research Hospital, Istanbul, Turkey

## Abstract

**Objectives:**

The inter-arm systolic blood pressure difference (IASBPD) is closely related to cardiovascular mortality and morbidity. The SYNTAX score indicates the extent and complexity of coronary artery disease, which are determined by coronary angiography. The aim of our study is to examine the relationship between the IASBPD (which is easily calculated in routine practice) and the SYNTAX score.

**Methods:**

104 patients were included in this cross-sectional study. The IASBPD was calculated by blood pressure measurements obtained simultaneously from both arms. The SYNTAX score was calculated by coronary angiography.

**Results:**

Patients were divided into two groups: those with a high SYNTAX score (≥20) and those with a low SYNTAX score (<20). The mean IASBPD values were significantly higher in the group with a high SYNTAX score (≥20) (p<0.001). The patients with IASBPD≥10 were more likely to have a high SYNTAX score compared to the patients with IASBPD<10 (p<0.001). Multiple logistic regression analysis revealed that only the IASBPD values were found to be independently associated with high SYNTAX score (OR: 1.717 (CI: 1.307-2.257), p<0.001).

**Conclusion:**

The IASBPD values obtained by only blood pressure measurements are closely related to the extent of coronary artery disease.

## 1. Introduction

The Synergy between PCI with TAXUS and Cardiac Surgery (SYNTAX) score is a commonly used tool for both short- and long-term risk classifications in patients undergoing percutaneous coronary intervention (PCI) [1, 2]. Although its prognostic value has been proven, it only provides an assessment on the image. It does not include clinical variables that are closely related to long- and short-term outcomes [3, 4]. However, the scores including clinical data require a lot of data and increase the risk of occurrence of incorrect results due to their complex structures.

The inter-arm systolic blood pressure difference (IASBPD) is associated with increased subclinical atherosclerosis in different areas of the arterial tree. According to a meta-analysis, inter-arm systolic blood pressure difference (IASBPD)≥10 mmHg was found to be related to cerebrovascular disease, vascular damage, and increased cardiovascular mortality [5]. It is a noninvasive and inexpensive indicator of arterial stiffness. It was found that the ankle-brachial index (ABI) showed both the extent of coronary artery disease and the post-PCI prognosis regardless of the SYNTAX score [6]. Studies have shown that blood pressure is not only related to narrowing of the coronary arteries but also affects the structure and content of atheromatous plaques [7]. However, IASBPD measurement can be simply applied to all patients without the need for Doppler. Moreover, hypertension (HT) guidelines recommend measuring blood pressure in both arms. In this study, we examined the relationship between the IASBPD and the SYNTAX score and evaluated the hypothesis that information can be received about the extent of coronary artery disease with a simple clinical instrument and thus about the prognosis.

## 2. Method

Our study had a cross-sectional study design. The IASBPD was calculated by obtaining blood pressure measurements in patients before coronary angiography (CAG). From among patients undergoing planned elective CAG because of the symptoms of ischemic heart disease and ischemic findings on the treadmill test or myocardial perfusion scintigraphy, 104 patients who met inclusion/exclusion criteria were included consecutively in the study. We excluded patients with a history of myocardial infarction, PCI, or coronary arterial bypass surgery. Patients with a history of congenital heart disease, severe valvular disease, cardiomyopathy, atrial fibrillation, impulse conduction disturbances or aortic coarctation, hemiplegia, and pulseless disease and those with history of transradial coronary intervention were not included. Informed consent was obtained from all patients. The study was approved by Institutional Ethics Committee and the investigation conformed to the principles of the Declaration of Helsinki.

### 2.1. Angiographic Analysis and Calculation of the SYNTAX Score

All patients underwent CAG through transfemoral approach. Coronary anatomy was examined by two experienced invasive cardiologists. As a result of these evaluations, the same opinion was reached. The SYNTAX score for each patient was calculated by scoring all coronary lesions with a stenosis diameter as 50% in vessels 1.5 mm, using the SYNTAX score algorithm, which is described in full elsewhere [8]. SYNTAX scores ≥20 were categorized as “high”, SYNTAX scores <20 were categorized as “low”. To calculate SYNTAX score II, the anatomical SS was combined with the following variables: age, creatinine clearance, LVEF, presence of ULM disease, peripheral vascular disease, female sex, and chronic obstructive pulmonary disease [4]

Height and body weight values were measured to calculate the Body Mass Index (BMI). HT was defined as systolic blood pressure ≥140 mm Hg and/or diastolic blood pressure ≥ 90 mm Hg or use of antihypertensive medication. Diabetes Mellitus was defined as fasting blood glucose level ≥126 mg/dL or use of insulin or an oral hypoglycemic medication. Coronary artery disease was assessed from patients' medical reports. Hyperlipidemia was defined by a total cholesterol of greater than 240 mg/dL and triglyceride of greater than 200 mg/dL. Current smoking was defined as smoking at least one cigarette per day in the year preceding the examination.

### 2.2. Measurement of Blood Pressure

The patients were made to sit for 5 minutes, with feet flat on the ground and back supported before BP measurements were taken. Two separate oscillometric blood pressure (BP) monitors with their own cuffs were used randomly, placed at the appropriate location on the arm and measured by the same trained nurse [9]. BP devices were validated by European Society of Hypertension (Omron HEM-7001-E; Omron Corp., Tokyo, Japan). An appropriate cuff was selected for each individual depending on their arm circumference. Simultaneous BP measurements were made three times by changing cuff and therefore BP monitor between arms each with 5 minutes interval. The IABPD was defined as the absolute difference between the blood pressure measurements in each arm [9]. Exaggerated IASBPD was defined as > 10 mmHg difference in systolic blood pressure (SBP) between two arms at all three measurements [10, 11]. Mean BP was calculated for both right arm and left arm using all 3 measurements. The difference was noted as the mean absolute IASBPD [9]. In order to evaluate reproducibility, BP measurements for 50 patients were performed by the same nurse. The intraclass correlation coefficient was calculated as 0.88 for BP difference between arms.

### 2.3. Laboratory Analyses

Blood collection was performed after a 12-hour fasting. All biochemical analyses were performed within the first two hours after blood collection. Glucose, creatinine, total cholesterol, high-density lipoprotein cholesterol, low-density lipoprotein cholesterol (LDL), and triglyceride levels were measured. Blood count analysis was performed using an automated cell counter (Sysmex XE-2100, Kobe, Japan).

### 2.4. Statistical Analysis

SPSS 17.0 for Windows (SPSS 17.0, Chicago, Illinois) software package was used in all analyses. The continuous variables were expressed as mean ± standard deviation (SD) (for parameters with normal distribution) and median (interquartile range, IQR) (for parameters without normal distribution), and categorical variables were expressed as percentages. The Chi-Square test was used to compare the categorical variables between the groups. Analysis of normality was performed with the Kolmogorov-Smirnov test. The independent samples* t-*test was used to compare continuous variables with normal distribution, and the Mann–Whitney* U* test was used to compare the continuous variables without a normal distribution. Correlations were sought with the Spearman's and Pearson correlation test. Binary logistic regression analysis (backward stepwise method) was performed to identify independently associated factors with high SYNTAX score (≥20). Variables with a **p** value <0.1 in univariate analysis were incorporated in the binary logistic regression analysis. Receiver-operating characteristic (ROC) curve analysis was performed to determine the optimum IASBPD cut-off value for predicting high SYNTAX score. A two-sided **p** value <0.05 was considered significant within a 95% confidence interval (CI).

## 3. Results

A total of 104 patients were included in our study. The mean age of the patients was 58.9 ± 9.5 years. 67 (64.4%) were male. While 99 (95.2%) patients had dominant right hand, the remaining 5 (4.8%) were left-handed. The mean SYNTAX value for all patients was 9.5 ± 8.2. In addition, 15 of the patients (14.4%) had a SYNTAX score ≥20.

When evaluated for the inter-arm blood pressure difference, the mean right arm systolic blood pressure (SBP) (p=0.012) and mean left arm systolic blood pressure (SBP) (p=0.019) and mean IASBPD values were significantly higher in the group with a high SYNTAX score (≥20) compared to the group with a low SYNTAX score (<20) (Table 1). The number of those with IASBPD≥10 was significantly greater in the group with a high SYNTAX score (≥20) compared to the group with a low SYNTAX score (<20) (12 (80.0%) versus 4 (4.5%), p<0.001). When biochemical data were examined, LDL levels were found to be higher in the group with a high SYNTAX score (≥20) compared to the group with a low SYNTAX score (<20) (152.3 (45.4) versus 125.4 (38.2), p=0.028).

Considering the relation between inter-arms blood pressure and SNYTAX II score, among the people whose SYNTAX II score are more than 20, mean of right arm SBP (135.1±19.3 versus 126.4±14.8 mmHg, p=0.020), mean value of left arm SBP (134.5±19.2 versus 122.7±17.0 mmHg, p=0.002), and mean IASBPD (10.6±6.6 versus 6.8±5.1 mmHg, p=0.003) values are measured higher.

Correlation was found between SYNTAX score and mean right arm SBP (r=0.333, p=0.001), IASBPD (r=0.736, p<0.001), and LDL levels (r=0.228, p=0.025).

Univariate regression analysis showed that LDL, IASBPD, gender, BMI, and exaggerated IASBPD (>10 mmHg) were significant at the p<0.1 level. However, since IASBPD and exaggerated IASBPD (>10 mmHg) are relative variables, the IASBPD values from these two variables were included in multiple logistic regression analysis. Multiple logistic regression analysis revealed that only the IASBPD values were found to be independently associated with high SYNTAX score (OR: 1.717 (CI: 1.307-2.257), p<0.001).

**Figure 1:**
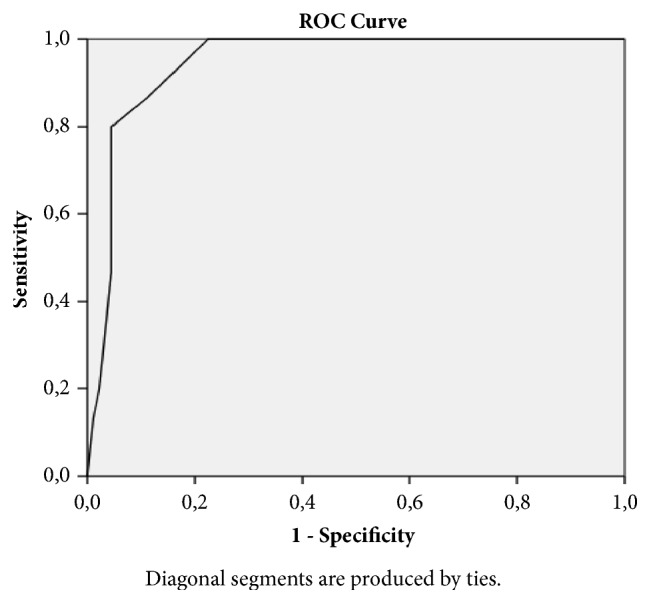
ROC (receiver-operating characteristic) curve in predicting the optimum inter-arm systolic blood pressure difference cut-off value for predicting high SYNTAX score.

The receiver-operating characteristic analysis showed that the optimal IASBPD cut-off value for predicting high SYNTAX score was 8.5 mmHg with a sensitivity of 86.0% and specificity of 89.1% (AUC = 0.94; 95% CI: 0.90-0.99) (Figure 1).

## 4. Discussion

In this study, we found a close association between the inter-arm blood pressure difference and the SYNTAX score (which indicates the severity and extent of coronary artery disease). We can obtain important data about the atherosclerosis severity of coronary artery disease and thus about the prognosis by blood pressure measurement in both arms, which is a simple and inexpensive method routinely recommended by HT guidelines.

Blood pressure measurement in both arms is recommended for each patient. It is important for determining the true value of blood pressure and thus the need for antihypertensive treatment especially in those with cardiovascular diseases. As in our study, simultaneous blood pressure measurement in both arms has a vital significance in determining the IASBPD [12]. Sequential measurements have a tendency to overestimate difference compared to simultaneous measurements. Recent studies have confirmed that the IASBPD is closely associated with cardiovascular mortality and peripheral arterial disease [9, 10]. The relationship between poor outcome and arterial stiffness in cardiovascular diseases has been demonstrated in different patient populations [13, 14]. The increase in arterial stiffness is systemic but often affects the arterial tree at different levels [14, 15]. Possibly, the changes in the amount of damage in elastic fibers are due to atherosclerosis at different levels in the arterial system, and the different aortic reflection wave responses cause significant IASBPD.

The SYNTAX score provides information on the choice of optimal revascularization type through coronary anatomy and on the short- and long-term prognosis in those undergoing coronary interventions. However, the SYNTAX score gives a prognosis according to only coronary anatomy. On the other hand, systemic atherosclerosis has proven to be closely associated with prognosis in those undergoing PCI [16]. It was seen that patients with peripheral arterial disease had a poor post-PCI prognosis and that combining the ankle-brachial index (ABI) and the SYNTAX score improved prognostic prediction [6, 17]. This demonstrates that evaluation of systemic atherosclerosis with the SYNTAX score improves prognostic prediction. Although the ABI is a noninvasive method, it is a more complex method than blood pressure measurement in both arms, which requires the use of Doppler ultrasound devices. The fact was that a close relationship between the IASBPD and the SYNTAX score was about the extent and complexity of coronary artery in our daily practice and about the prediction of PCI results with additional studies. Although the clinical SYNTAX score including clinical variables gives more clear prognostic information than the classic SYNTAX score, the clinical SYNTAX score includes complex calculation methods. The IASBPD is easy and simple to measure and is a convenient method for daily practice.

## 5. Limitations

Our study has some limitations. The number of the patients was relatively low. It was conducted in a single center. Since the SYNTAX score is relatively low, cut-off values may be different for groups with complex and diffuse coronary artery disease. Routine radiological evaluation was not performed for the aorta and its branches in those with IASBPD>10 mmHg. However, performing radiological examinations in all these patients is not appropriate in terms of both time and cost. In addition, most of these patients do not have pathologies such as anomaly, arthritis, and aneurysm in their arterial system [18]. Power analysis was not performed to calculate the sample size.

## 6. Conclusion

We found that the IASBPD that would be obtained by a simple and easy blood pressure measurement was associated with the extent of coronary artery disease obtained by coronary angiography. This situation, which is a reflection of systemic involvement of atherosclerosis, gives information about the extent of coronary artery disease in patients and also may provide us with information on prognosis as a result of prospective studies to be performed.

## Figures and Tables

**Table 1:** Comparison of groups with high and low SYNTAX scores in terms of demographic and biochemical data.

**Variables**	**SYNTAX≥20**	**SYNTAX<20**	**p**
	**N=15**	**N=89**	
**Age (years)**	60.7 (11.0)	58.6 (9.3)	0.433

**Gender (male) (n, **%**)**	13 (%86.7)	54 (%60.7)	0.052

**Right-hand dominance **	12 (%85.7)	81 (%96.4)	0.092
**(n, **%**)**			

Diabetes mellitus	4 (%26.7)	38 (%42.7)	0.242
**(n, **%**)**			

Hypertension	6 (%40.0)	50 (%56.2)	0.245
**(n, **%**)**			

Hyperlipidemia** (n, **%**)**	3 (%20.0)	23 (%25.8)	0.629

**Smoking (n, **%**)**	5 (%35.7)	28 (%31.8)	0.776

**BMI **(kg/m^2^)	28.9 (3.6)	29.1 (6.8)	0.085

**Mean of right arm SBP (mmHg)**	134.4±12.6	124.4±14.4	**0.012**

**Mean value of left arm SBP (mmHg)**	133.4±9.7	124.6±13.7	**0.019**

**Mean value of right arm **	74.6±5.0	72.9±10.4	0.562
**DBP (mmHg)**			

**Mean value of left arm **	73.6±15.7	72.0±13.1	0.682
**DBP (mmHg)**			

**Mean IASBPD (mmHg)**	11.6±2.3	5.5±2.6	**<0.001**

**Mean IADBPD (mmHg)**	5.3 ±1.7	4.2±2.9	0.188

**Exaggerate IASBPD **	12 (%80)	4 (%4.5)	**<0.001**
**(>10 mmHg)**			

**Glucose **(mg/dL)	120.6 (23.9)	122.9 (34.9)	0.816

**TC** (mg/dL)	207.4 (70.6)	195.0 (50.0)	0.410

**LDL-C **(mg/dL)	152.3 (45.4)	125.4 (38.2)	**0.028**

**HDL-C **(mg/dL)	43.3±9.2	44.2 ±11.7	0.993

**Triglyceride** (mg/dL)	174.0±17.0	162.3±21.2	0.773

**Creatinine **(mg/dL)	0.8±0.1	0.7±0.2	0.819

**SYNTAX score**	27.3±3.1	6.5±3.9	**<0.001**

Variables that fit a normal distribution were expressed as mean ± SD. Variables that did not fit a normal distribution were expressed as median (interquartile range).

BMI: body mass index, DBP: diastolic blood pressure, HDL-C: high-density lipoprotein cholesterol, IASBPD: inter-arm systolic blood pressure difference, IADBPD: inter-arm diastolic blood pressure difference, LDL-C: low-density lipoprotein cholesterol, SBP: systolic blood pressure, TC: total cholesterol.

## Data Availability

The data used to support the findings of this study are available from the corresponding author upon request.
